# Serotonin 2A receptors contribute to the regulation of risk-averse decisions

**DOI:** 10.1016/j.neuroimage.2013.06.063

**Published:** 2013-12

**Authors:** Julian Macoveanu, James B Rowe, Bettina Hornboll, Rebecca Elliott, Olaf B Paulson, Gitte M Knudsen, Hartwig R Siebner

**Affiliations:** a Danish Research Centre for Magnetic Resonance, Copenhagen University Hospital, Hvidovre, Denmark; b Center for Integrated Molecular Brain Imaging, Copenhagen University Hospital, Copenhagen, Denmark; c Department of Clinical Neurosciences, Cambridge University, Cambridge, UK; d Neuroscience and Psychiatry Unit, University of Manchester, Manchester, UK; e Neurobiology Research Unit, Copenhagen University Hospital, Rigshospitalet, Copenhagen, Denmark

**Keywords:** Serotonin, Ketanserin, Pharmacological fMRI, Reward, Decision-making, Frontopolar cortex

## Abstract

Pharmacological studies point to a role of the neurotransmitter serotonin (5-HT) in regulating the preference for risky decisions, yet the functional contribution of specific 5-HT receptors remains to be clarified. We used pharmacological fMRI to investigate the role of the 5-HT_2A_ receptors in processing negative outcomes and regulating risk-averse behavior. During fMRI, twenty healthy volunteers performed a gambling task under two conditions: with or without blocking the 5-HT_2A_ receptors. The volunteers repeatedly chose between small, likely rewards and large, unlikely rewards. Choices were balanced in terms of expected utility and potential loss. Acute blockade of the 5-HT_2A_ receptors with ketanserin made participants more risk-averse. Ketanserin selectively reduced the neural response of the frontopolar cortex to negative outcomes that were caused by low-risk choices and were associated with large missed rewards. In the context of normal 5-HT_2A_ receptor function, ventral striatum displayed a stronger response to low-risk negative outcomes in risk-taking as opposed to risk-averse individuals. This (negative) correlation between the striatal response to low-risk negative outcomes and risk-averse choice behavior was abolished by 5-HT_2A_ receptor blockade. The results provide the first evidence for a critical role of 5-HT_2A_ receptor function in regulating risk-averse behavior. We suggest that the 5-HT_2A_ receptor system facilitates risk-taking behavior by modulating the outcome evaluation of “missed” reward. These results have implications for understanding the neural basis of abnormal risk-taking behavior, for instance in pathological gamblers.

## Highlights

• Blocking serotonin 5-HT_2A_ receptors renders healthy subjects more risk-averse.
• It also reduces lateral frontopolar cortex response to low-risk aversive outcomes.
• The striatal response to low-risk aversive outcomes correlates with risk preference.
• 5-HT_2A_ receptor blockade abolished this correlation.

## Introduction

When choosing between risky alternatives, people take into account both probabilities and valuation (utility) of possible outcomes, considering probability-weighted expectation over possible utilities (Von Neumann et al., 2007). For risky financial decisions, people's choice behavior shows systematic deviations from the standard economic view of expected utility maximization (Rieskamp, 2008). For instance, when playing lotteries, excessive “decision weights” are assigned to low-probability outcomes (Hsu et al., 2009). Such non-linear attributes that bias objective probabilities have been implemented in models of behavioral economics such as the prospect theory (Kahneman and Tversky, 1979), yet it is only recently that researchers have started to explore its neural underpinnings (e.g. Christopoulos et al., 2009, Huettel et al., 2006). Unveiling the neural mechanisms that bias the “decision weights” of risk-taking behavior is critical to the understanding of pathological states of impaired financial decision-making such as gambling addiction.

The availability of striatal dopamine D1 receptors has recently been shown to be negatively correlated with the degree of nonlinearity of the risk weighting function (Takahashi et al., 2010). A reduced striatal D1 receptor density was associated with a more pronounced overestimation of low winning probabilities and underestimation of high winning probabilities. Genetic differences in striatal dopamine availability have also been proposed to explain differences in individuals risk taking behavior (Mata et al., 2012).

The neurotransmitter serotonin (5-HT) may also play a critical role in biasing the “decision weights” guiding risky behaviors. 5-HT has been implicated in processing and learning from aversive stimuli (Tanaka et al., 2009) as well as in the prediction of future punishment (Boureau and Dayan, 2011, Crockett et al., 2012). Acute reduction of central serotonin levels with acute dietary tryptophan depletion (ATD) increases the propensity to make risky choices in a gambling task both in humans (Rogers et al., 1999a, Rogers et al., 1999b) and non-human primates (Long et al., 2009). In healthy individuals, tryptophan supplements altered the weighting of gains and small losses and reduced the reflection effect (being risk-averse for gains and risk-seeking for losses) (Murphy et al., 2009). Despite the clear involvement of 5-HT in regulating risk-taking behavior the functional contribution of specific 5-HT receptors remains to be clarified.

The 5-HT_2A_ receptors have excitatory effects on the postsynaptic neurons and are abundantly and uniformly distributed, primarily in cortical, but also in subcortical regions (Van Dyck et al., 2000). Interestingly, 5-HT_2A_ receptor stimulation increases activity in the nigrostriatal dopamine pathway modulating phasic, but not tonic, dopamine efflux (Alex and Pehek, 2007). Impulsive behavior, a dominant feature in pathological gamblers, has been attributed to a polymorphism (1438A/G) in the promoter of the 5-HT_2A_ receptor gene (Nomura et al., 2006) and recent imaging studies have linked frontolimbic 5-HT_2A_ binding to neuroticism (Frokjaer et al., 2008). Prefrontal 2A receptors have also been implicated in regulating the amygdala–prefrontal coupling during aversive face processing (Fisher et al., 2009). In order to explore the role of 5-HT_2A_ receptor-mediated neurotransmission in regulating risk behavior we used functional magnetic resonance imaging (fMRI) in healthy volunteers during the performance of a gambling task while blocking 5-HT_2A_ receptor function with ketanserin.

Compared to a state with normal 5-HT_2A_ receptor function, we expected ketanserin to alter the response in several regions that are modulated by serotonergic projections and that play key roles in reward processing (Liu et al., 2011). For instance, the lateral frontopolar cortex (lFPC) has been linked to choices between options associated with different risks and reward magnitudes (Rogers et al., 1999b) and to tracking missed reward (Boorman et al., 2009, Boorman et al., 2011). Goal-directed behavior and decision-making are known to rely on frontopolar–striatal projections (Haber, 2011). During primary rewarding stimuli, these fronto–striatal interactions are sensitive to serotonergic drugs (Abler et al., 2012). Global serotonergic challenges have recently been found to modulate the activity of dorsomedial prefrontal cortex and amygdala when subjects missed a large monetary reward (Macoveanu et al., in press). Increased 5-HT availability by selective serotonin uptake inhibitors (SSRIs) has been reported to reduce the neural response of ventral striatum (VS) to primary rewarding stimuli (Abler et al., 2012, McCabe et al., 2010). Given the relevance of 5-HT for processing and predicting aversive stimuli (Boureau and Dayan, 2011, Cools et al., 2010, Crockett et al., 2012), we predicted that blocking the excitatory action of the 5-HT_2A_ receptors would modulate the neural response in reward-related regions while subjects evaluate monetary outcomes, thereby altering risk-taking behavior.

## Patients and methods

### Participants

Twenty right-handed healthy adults (7 females) age ± SD of 32.1 ± 5.9 years were included in the fMRI study. None reported a history of stimulant abuse, neurological or psychiatric disorders. All subjects were naïve for antipsychotics and antidepressants according to self-report. Written informed consent was obtained prior to the MRI scanning sessions. The study was approved by the Copenhagen Ethics Committee (KF 01-2006-20).

### Card gambling task

**Fig. 1:**
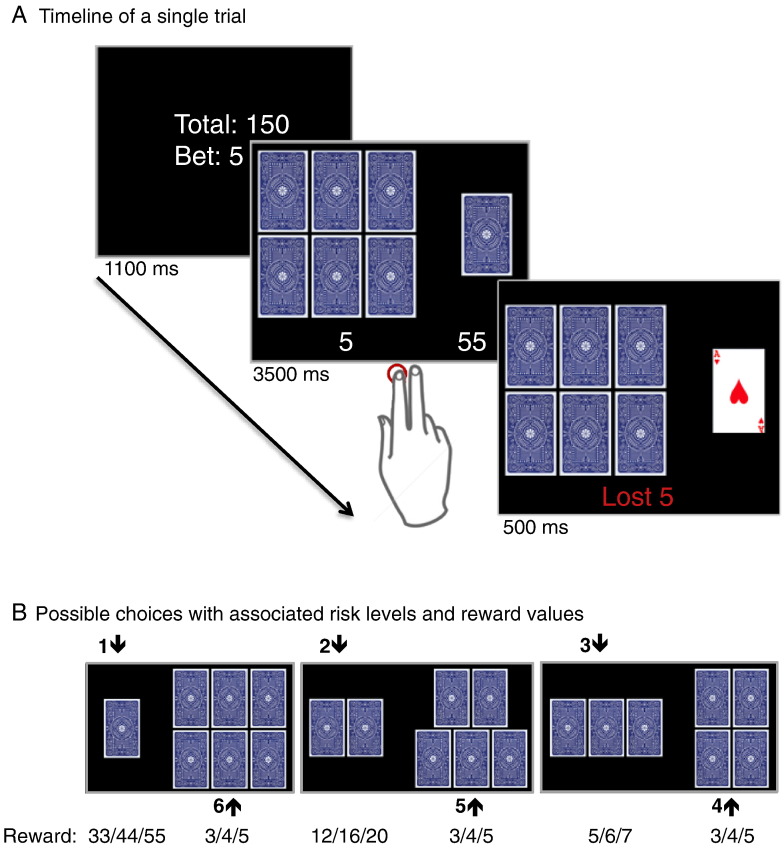
The card gambling task. (A) Temporal structure of a single gambling trial. Each trial was divided into three phases: INFORMATION, DECISION and OUTCOME. Subjects first received INFORMATION about the sum of money they had accumulated and the bet size (3, 4 or 5 DKK), which could be lost. In the DECISION phase, two sets of cards facedown were presented together with the associated monetary reward. Participants chose the set of cards where they believed the “ace of hearts” would be hidden. In the OUTCOME phase, the “ace of hearts” was revealed, providing the subjects a feedback whether they chose the right set and won the associated reward or lost the bet. (B) The six possible choices with associated winning amounts in DKK.

During fMRI, participants performed a gambling task previously described in detail in Macoveanu et al. (in press). Each trial started with an information screen displaying the total amount of money available in Danish Kroner (1$ ≈ 6 DKK) and the bet size. Seven playing cards were randomly distributed into two sets displayed face down (Fig. 1A). One of the cards was the “ace of hearts” and subjects were asked to choose one of the two sets that they believed contained the ace. Upon selection, the location of the ace was revealed. A correct choice was rewarded with the amount displayed bellow the set. For a wrong choice the subject would lose the bet. Fig. 1B shows the six possible risk choices with associated potential rewards. The experimental design enabled us to assess differential responses to outcomes depending on whether the decision preceding it was risk-averse or risk-taking. The paradigm equated the expected values of the high and low risk choices (i.e., the sum of probabilistically weighted wins and losses) and was tuned towards wining and therefore more sensitive to risk avoidance (Ludvig and Spetch, 2011). Subjects performed the paradigm in two sessions with a one-minute break in-between. Each of the two sessions lasted for 11 min and included different randomizations of 28 choices between one and six cards, 28 choices between two and five cards, 28 choices between three and four cards and 28 null events of the same length as a real event where a fixation cross was presented instead of the task screen. The highest final amount of the two sessions was paid to the participants (average 203 ± 54 DKK).

### Serotonergic challenge

Participants were assigned in a randomized counterbalanced fashion to scanning under each of four conditions: three different serotonergic challenges and one session without any pharmacological treatment referred to as “control session”. The three serotonergic challenges consisted of (i) acute blockade of 5-HT_2A_ receptors with ketanserin, (ii) acute tryptophan depletion (ATD) to decrease tryptophan availability in the brain, and (iii) intravenous administration of the SSRI citalopram to acutely increase levels of free serotonin in the brain. Sessions were performed at least one week apart to ensure a proper wash-out, using an identical fMRI protocol that included three fMRI runs during which participants engaged in a card gambling task, a NoGo task, and an implicit face emotion task. Here we only report the results obtained during the card gambling task. The order of 5-HT challenges and fMRI runs was fully counterbalanced across subjects to control for task and scan repetition effects. The citalopram and ATD interventions addressed the opposing effects of increased versus decreased serotonin levels in the brain, and these results have been recently reported in a separate paper (Macoveanu et al., in press). The main purpose of the present study was to explore the specific function of the 5-HT_2A_ receptors by comparing behavior and brain responses while the 5-HT_2A_ receptors were blocked using ketanserin as opposed to the control session.

In the ketanserin session, ketanserin was administrated intravenously, starting with a 10 mg bolus followed by 6 mg/h maintenance rate during the entire length of the functional image acquisition (~ 17.5 mg ketanserin in total). The interval between the bolus and initiation of the fMRI paradigm ranged from 4 to 69 min across subjects. We estimated the individual average 5-HT_2A_ receptor occupancy rates across the duration of the paradigm to range from 82% to 100%. The estimation was performed based on 5-HT_2A_ occupancy data from our previous ^18^F-altanserin PET study that used ketanserin infusion and which allowed calculation of the time course of the ketanserin binding and liberation of radioligand from the 5-HT_2A_ receptors (Pinborg et al., 2003). We measured the blood pressure every 10 min during the entire session. None of the subjects developed hypotension during the intravenous ketanserin challenge.

The ATD protocol, which required 24 h of protein diet and ingestion of an amino acid mixture lacking tryptophan 5 h prior to the MRI investigation (Macoveanu et al., in press), differed substantially from the intravenous infusion protocols used to administer citalopram and ketanserin. For practical reasons with respect to subjects' compliance, we decided against the intravenous and oral administration of drug and placebo across all four separate scanning sessions. We wish to emphasize that our experimental design still enabled us to assess non-specific effects related to discomfort or distress during intravenous drug administration because the experiment comprised two intravenous challenges involving intravenous drug administration during the entire MRI scan. We reasoned that non-specific effects related to the intravenous ketanserin challenge on behavior and neural activity should also be evident in the data acquired during the citalopram session, whereas 5-HT_2A_ receptor-specific effects should only be present in the ketanserin session. As a post-hoc analysis we validated the observed changes induced by ketanserin compared to the control session by comparing ketanserin against the citalopram session.

Note that although the pharmacological procedures were not equivalent across the experimental sessions, participants were not given any information about potential effects of the drugs that were given. While subjects were informed of the possible side effects arising from study participation, they were not informed about which drug they would be receiving on a specific day or about the differences in probabilities of side effects between the different drug interventions or the expected effects of the 5-HT manipulations.

### Behavioral data analysis

The frequency of risk choices and reaction times were entered into repeated measures analyses of variance (rmANOVA) models (PASW-SPSS17 Statistics software, Chicago) with fixed factors of type of intervention (2 levels, ketanserin and control) and “risk level” (3 levels, odds of 4/7, 5/7 and 6/7) as within subject factors. In order to avoid including perfectly collinear data, we examined the frequencies of the 4/7, 5/7 and 6/7 choices only. Each of these lower risk options was paired to a corresponding higher risk option in a forced-choice design (Fig. 1B). We further set up a post-hoc analysis to test whether the individual risk preference in a trial (evaluated as the rate of risk choices with odds smaller than 50%) was influenced by the risk level (all 6 levels) and outcome of the immediately preceding trial (negative or positive). For this we used an rmANOVA with 3 factors: intervention (control and ketanserin), risk (6 levels), and outcome (negative and positive). Significance threshold was set at p < 0.05 using the Greenhouse–Geisser correction for non-sphericity when appropriate. Conditional on significant F-values, pair-wise post-hoc t-tests were performed to further explore significant main effects and interactions.

### MRI data acquisition

All MRI measurements were performed on a 3 Tesla MR scanner (Siemens Trio, Erlangen, Germany) using an eight-channel head array coil. The same MRI protocol was performed during the control, ketanserin and citalopram sessions. BOLD-sensitive fMRI used a T2*-weighted gradient echo spiral echo-planar imaging (EPI) sequence with a repetition time (TR) of 2.5 s, echo time (TE) of 26 ms, and flip angle of 90°. The fMRI measurements were obtained in two fMRI runs, each run lasting 11 min. A total of 260 brain volumes were acquired in a single fMRI session. Each brain volume consisted of 41 slices with a slice thickness of 3 mm, between-slice gap of 25%, and a field of view (FOV) of 192 × 192 mm using a 64 × 64 grid. The EPI sequence was optimized for signal recovery of frontal cortex close to the base of the skull by tilting slice orientation from a transverse towards a coronal orientation by about 30° and the use of a preparation gradient pulse (Deichmann et al., 2003). In addition, high-resolution 3D structural T1-weighted spin echo images were obtained after the first session of BOLD fMRI (TI = 800, TE = 3.93, TR = 1540 ms, flip angle 9°; 256 × 256 FOV; 192 slices). After the BOLD fMRI measurements, we assessed regional blood perfusion at rest using Arterial Spin Labeling (ASL). The ASL measurements were performed to test whether any differences in the regional BOLD signal between ketanserin and control sessions resulted from a real difference in regional neural activity induced by 5-HT challenge rather than a mere difference in baseline blood perfusion levels. ASL-based perfusion measurements used FAIR Q2TIPS (Luh et al., 1999) sequences with 3D GRASE (Günther et al., 2005) single-shot readout with background suppression (TR = 3.4 s, TE = 19.3 ms, TI = 200, 400, 600, 800, 1000, 1200, 1400, 1600, 1800, 2000, 2200, 2400, 2600, 2800, 3000 ms, 2 averages per TI, Q2TIPS saturation duration = 150 ms, 26 slices, voxel size = 5.0 × 5.0 × 4.0 mm, FOV = 320 × 160 × 104 mm, vessel suppression with bipolar gradients, b = 6 s/mm2). The duration of the ASL measurements was 4 min and the sequence was run right after the fMRI session. Pulse and respiration were recorded during the entire MRI session.

### fMRI data analysis

The preprocessing and statistical analysis of the acquired images was done using SPM5 (http://www.fil.ion.ucl.ac.uk/spm/software/spm5). The structural images were segmented and the resulting parameters were used during the normalization of the functional images. The functional images were realigned to the first image, normalized and smoothed using a symmetric 8-mm Gaussian kernel.

We implemented two event related 1st level subject models, one for the decision phase and one for the outcome phase of the gambling task. The decision phase statistical model included six regressors of interest, one for each risk level (from the lowest odds 1/7, to the highest odds 6/7). The different choices are illustrated in Fig. 1B. The model also included one regressor for outcome and one for the information phase. We identified brain regions showing an interaction between risk level and type of challenge by including the six 1st level contrasts of interest in a 2nd level ANOVA model with three factors: “subject” (20 levels), “type of pharmacological challenge” (2 levels: ketanserin and control) and “risk level” (6 levels: 1/7 to 6/7).

The outcome phase 1st level models included negative and positive outcome regressors separated by the risk level, one decision and one information phase regressors. Due to interindividual differences in risk preference, in order to have enough measurements for all types of outcome events, the negative and positive outcomes were grouped: choices with odds of 1/7 and 2/7 were modeled together as a “high-risk” contrast, choices with odds of 3/7 and 4/7 as a “medium-risk” contrast and choices with odds of 5/7 and 6/7 as a “low-risk” contrast. Thus, the 1st level outcome models resulted in six contrasts of interest used for the group level analysis: three negative and three positive outcome contrasts depending on the risk level. In order to identify brain regions where ketanserin had an effect on either negative or positive outcome related activity depending on the risk level of the decision causing the outcome, we set up a 2nd level ANOVA model using the negative outcome 1st level contrasts and a second analog model with the positive outcome 1st level contrasts. The ANOVA models included three factors: “subject” (20 levels), “type of pharmacological challenge” (2 levels: ketanserin and control) and “risk level” (3 levels: high-, medium-, low-risk). These models were used to assess the main effects of positive and negative outcomes and to test for interactions between the riskiness of choice behavior and pharmacological challenge. We evaluated the direction of the intervention effect by separately comparing individual risk level contrasts of the two challenges.

Differences in response to positive and negative outcomes, as well as commonly activated regions (conjunction analysis) were assessed in a separate 2nd level ANOVA model that included an “outcome” factor (2 levels: positive and negative), a “risk level” factor (3 levels: high-, medium-, low-risk) and “type of pharmacological challenge” factor (2 levels: control and ketanserin).

Because the outcome phase always followed the decision phase and we wanted to assess regions uniquely involved during the outcome phase we controlled for the carryover effect of the BOLD response during the decision phase onto the outcome phase by exclusively masking the outcome contrasts with the decision contrast at p < 0.001 uncorrected. In addition to the contrasts of interest, all 1st level models also included 40 additional nuisance regressors to correct for physiological noise related to pulse (10), respiration (6) and movement (24) (Glover et al., 2000, Lund et al., 2006).

We were also interested in assessing interaction effects between ketanserin-induced changes in behavior and brain response. For this purpose we set up a 2nd level linear regression model that included the negative outcome low-risk contrasts from the control and ketanserin groups together with the frequency of low-risk choices as covariates. The low-risk contrast images were kept separate for 5/7 and 6/7 odds. However, as a few subjects lacked the 6/7 low-risk negative outcome event we controlled for a possible bias in subject contributions to the data by verifying the results in a separate model where the 5/7 and 6/7 low-risk events were pooled together. The significance of the linear relationship between BOLD response and choice behavior was assessed individually for the control and ketanserin sessions using linear regression models implemented in SPM.

To control for possible non-specific effects of the drug's administration protocol and indirect effects of drug we rerun all 2nd level models with the control session substituted by the citalopram session.

We considered clusters to be significant at p = 0.05 after Family-Wise Error (FWE) correction for multiple non-independent comparisons applying a cluster-extent threshold of p < 0.005. We also present exploratory results for contrasts of interest at a reduced voxel-wise threshold, uncorrected p < 0.001. We report small volume corrections (SVC) for brain regions for which we had a priori hypothesis (see Introduction). For this end we defined spherical regions of interest (ROIs) with a diameter of 8 mm around lFPC peaks (MNI x,y,z = − 34, 56, − 8 and 36, 54, 0) from Boorman et al. (2009) and VS peaks (MNI x,y,z = − 8, 14, − 4 and 6, 14, − 8) from McCabe et al. (2010). The significant clusters are reported with Z-score and stereotactic MNI coordinates of the regional maxima.

### Mood assessment

Participants completed a modified Danish version of the Profile of Mood States (POMS) questionnaire (McNair et al., 1971) to assess current mood according to six domains: tension/anxiety, depression/dejection, anger/hostility, vigor/activity, fatigue/inertia and confusion/bewilderment. For both the control and ketanserin sessions, participants completed the mood questionnaire twice at each session: upon arrival and immediately after the fMRI scan.

### Cerebral blood perfusion analysis

The ASL-based brain perfusion measurements were analyzed using FABBER with spatial priors (http://www.fmrib.ox.ac.uk/fsl/fabber). Perfusion differences between control and ketanserin sessions were evaluated using permutation-based statistics. We made two perfusion contrasts at an extended threshold of p < 0.01 uncorrected for multiple comparisons for increased and decreased perfusion levels between the control and ketanserin sessions. The two contrasts were tested as exclusive masks for the BOLD contrasts in order to exclude regions showing perfusion changes from contrasts with BOLD changes induced by ketanserin.

## Results

### Behavioral results

**Fig. 2:**
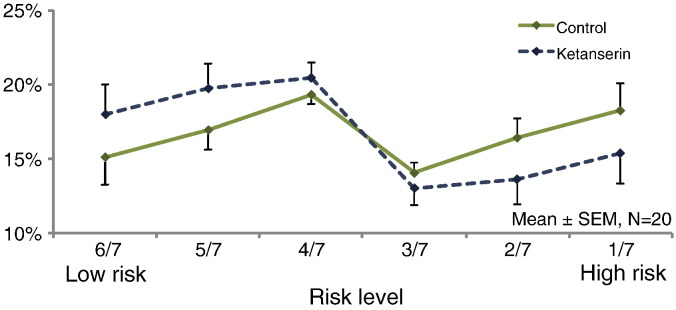
Distribution of risk choices across the six different risk levels. Ketanserin led to a significant increase in the frequency of low-risk choices.

As intended, participants distributed the selection of choices relatively evenly across the six risk levels (Fig. 2). Acute blockade of the 5-HT_2A_ receptor resulted in a shift towards more risk-averse choices. Participants made significantly more low-risk choices during the ketanserin session compared to the control session (F1,19 = 5.3, p < 0.05). An additional ANOVA addressing the dependence of risk preference on the risk level and outcome of the immediately preceding trial again showed a main effect of drug intervention with less high-risk choices in the ketanserin session (F1,19 = 4.60, p < 0.05) and a main effect of risk level (F3,62 = 4.6, p < 0.01). There was also an interaction between risk level and the outcome (negative or positive) of the preceding trial (F4,75 = 8.1, p < 0.001): both, losing a low-risk bet or winning a high-risk bet, facilitated risk averse decisions in the next trial. Conversely, winning a low-risk bet and losing a high-risk bet prompted subjects to make more often risky decision in the next trial. This recency effect on risk-taking was not modulated by ketanserin.

Reaction times were not influenced significantly by the ketanserin challenge. There was however significant influence of risk level on reaction times (F(3,58) = 5.1, p < 0.01). A post-hoc analysis revealed a significant quadratic effect of risk level. Decisions between similar risk choices (3/7 and 4/7) were associated with the longest reaction times, presumably due to a higher ambiguity of this choice condition. This inverse U-shaped effect was significant only for the control session (F(2,12) = 3.2, p < 0.05).

### Neural response to negative outcomes

**Fig. 3:**
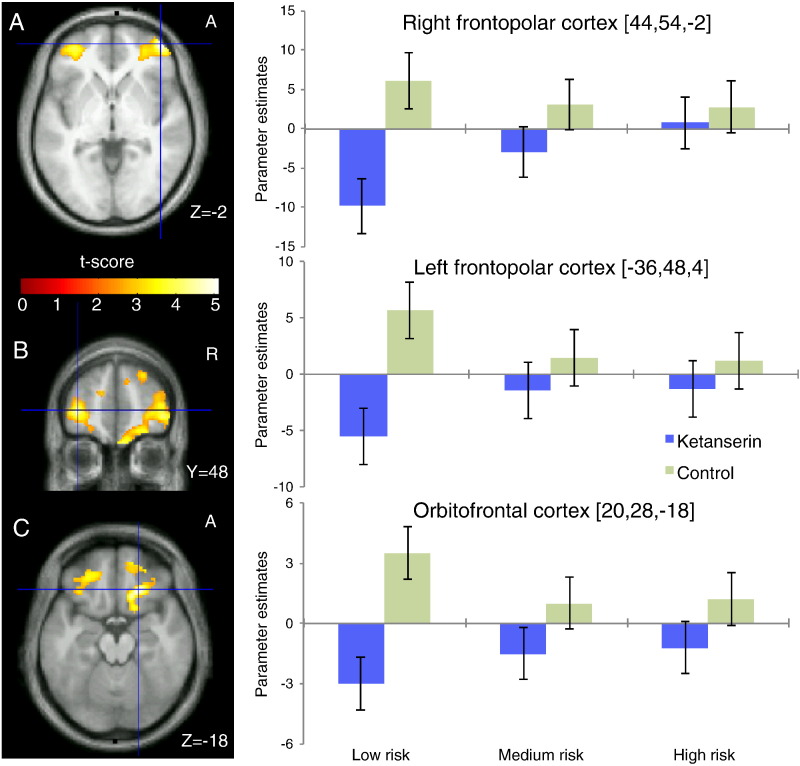
Bilateral reductions in the neural response to negative outcomes caused by low-risk decisions in lateral frontopolar cortex after 5-HT_2A_ blockade. Choices with odds of 1/7 and 2/7 were pooled together as high-risk, choices with odds of 3/7 and 4/7 as medium-risk and choices with odds of 5/7 and 6/7 as low-risk. The figure shows the control > ketanserin contrast for low-risk choices. Left panels, for low-risk negative outcomes, ketanserin decreased right and left lateral frontopolar cortex activity compared to the control condition. The maps are thresholded at p < 0.01 (uncorrected) for illustrative purposes. The right panels give the parameter estimates of the regional peaks for the negative outcomes caused by low, medium and high-risk choices and serotonergic challenge. Error bars represent 90% confidence interval of the mean.

Negative outcomes (i.e. losing a bet) independent of risk level and drug intervention consistently increased neural activity as indexed by the BOLD response in a bi-hemispherical network encompassing the medial frontal cortex, lateral orbitofrontal cortex (OFC), anterior insular cortex, lateral prefrontal and frontopolar cortex (Table 1). The group-by-risk ANOVA analysis yielded a significant interaction between the risk level (low vs. high) and the effect of ketanserin on the BOLD response to negative outcomes in the right lFPC ([42 56 − 2], Z = 3.3, p_svc_ = 0.04) and right VS [10 14 − 14], Z = 3.3, p_svc_ = 0.05), indicating a differential effect of ketanserin on processing negative outcomes depending on the risk level. Post-hoc tests revealed that 5-HT_2A_ receptor blockade by ketanserin attenuated the BOLD response to low-risk negative outcomes in lFPC bilaterally and ventral regions of the right OFC (Fig. 3, Table 2). Noteworthy, this effect was found significant only for negative outcomes caused by low-risk decisions that resulted in a high missed reward, and not for negative outcomes caused by medium-risk and high-risk decisions. The interaction in right VS was driven by both a non-significant attenuation of the response to low-risk negative outcomes and a non-significant increase of the response to high-risk negative outcomes during the ketanserin session.

**Table 1:** Significant cluster peaks from the main effect of negative outcomes (vs. baseline), positive > negative outcomes (see also Fig. 5A) and decision phase analyses across all risk levels. Voxels are thresholded at p < 0.05 FWE corrected (cluster minimum 10 voxels, sub-peaks separated by > 20 mm). Coordinate x,y,z values in MNI standard stereotactic space, and Z statistics.

Region	Side	x	y	z	Z-stat
*Negative outcomes*					
Frontopolar cortex	Left	− 40	52	− 4	6.7
	Right	42	48	− 2	> 8
Lateral orbitofrontal cortex	Left	− 44	26	− 8	> 8
	Right	47	26	− 8	7.8
Anterior insula	Left	− 30	16	− 14	> 8
	Right	34	14	12	> 8
Inferior parietal cortex	Left	− 58	− 50	36	> 8
	Right	56	− 48	42	> 8
Middle frontal gyrus	Left	− 38	10	50	6.8
	Right	42	10	46	> 8
Caudate	Left	− 8	0	4	6.7
	Right	10	0	6	> 8
Medial frontal cortex	Both	− 2	44	34	> 8

*Positive outcomes > negative outcomes*					
Ventral striatum	Left	− 16	4	− 12	7.3
	Right	16	6	− 14	7.6
Precuneus	Left	− 20	− 66	32	5.9
	Right	12	− 56	22	5.5
Posterior cingulate cortex	Both	− 4	− 38	38	5.7

*Decision phase (increased response with high risk)*					
Ventral striatum	Left	− 10	10	− 8	5.3
	Right	16	4	− 12	5.0
Inferior parietal lobe	Left	− 38	− 42	56	4.9

**Table 2:** Peak differences between low-risk negative outcome contrasts from the Control vs. Ketanserin and Citalopram vs. Ketanserin analyses (clusters surviving p < 0.05 FWE correction, sub-peaks separated by > 20 mm). Coordinate x,y,z values in MNI standard stereotactic space, and Z statistics.

		Control > Ketanserin				Citalopram > Ketanserin			
Region	Side	x	y	z	Z-stat	x	y	z	Z-stat
Frontopolar cortex	Left	− 36	48	4	4.0	− 36	52	2	3.8
	Right	44	54	− 2	4.5	44	54	− 2	3.4
Orbitofrontal cortex	Right	20	28	− 18	4.5	14	26	− 18	3.8

Correlation with risk preference		Ketanserin > Control				Ketanserin > Citalopram			

Ventral striatum	Left	− 14	6	− 12	3.9	n/a			
	Right	16	8	− 14	4.2	16	10	− 16	3.5
Superior frontal sulcus	Right	18	8	46	4.0	n/a			

**Fig. 4:**
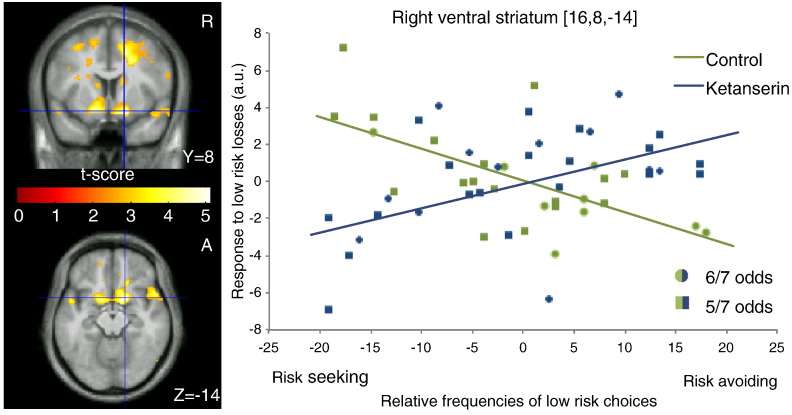
Change in the correlation between the regional response to low-risk negative outcomes and risk-averse behavior after 5-HT_2A_ blockade. The left panels display regions showing differential correlations (control > ketanserin) between the response to low-risk negative outcomes and mean normalized frequency of low risk choices (5/7 and 6/7), maps thresholded at p < 0.01 (uncorrected) for illustrative purposes. Right panel shows the plot of the linear regressions for the control and ketanserin groups in the regional peak of ventral striatum.

The 2nd level linear regression model testing the individual choice preference (indexed by the frequency of low-risk choices with odds of 5/7 and 6/7) as a function of BOLD response to negative outcomes revealed a significant group-by-behavior interaction in VS (Fig. 4). In a state of normal 5-HT_2A_ receptor function (control condition), the VS showed a stronger BOLD response to low-risk negative outcomes in those subjects who relatively frequently chose high-risk options (i.e., bets with an odds of 1/7 or 2/7). Conversely, subjects who made more low-risk choices showed a weaker VS response when these low-risk choices turned out to be unsuccessful (Fig. 4, right panel). The ketanserin induced change in the slope of the linear relationship between the BOLD response to negative outcomes and risk preference was significant in both right and left VS and right superior frontal sulcus (Table 2). Because some of the subjects lacked either the 5/7 or 6/7 low-risk negative outcome event, the analysis was repeated with pooled 5/7 and 6/7 events. The additional analysis was consistent with the initial one showing a trend interaction between the effect of ketanserin and frequency of low-risk choices in right VS ([8 14 − 16], Z = 3.0, p = 0.001).

A post-hoc linear regression analysis between the baseline BOLD response to negative outcomes (control session) and relative frequency of low-risk choices (5/7 and 6/7) yielded a negative correlation in right VS ([18 12 − 2], Z = 3.4, p < 0.001 uncorrected) and left VS ([− 14 0 − 10], Z = 3.9, p < 0.001 uncorrected). The analog analysis for the ketanserin session did not yield a significant correlation in VS.

### Neural response to positive outcomes

**Fig. 5:**
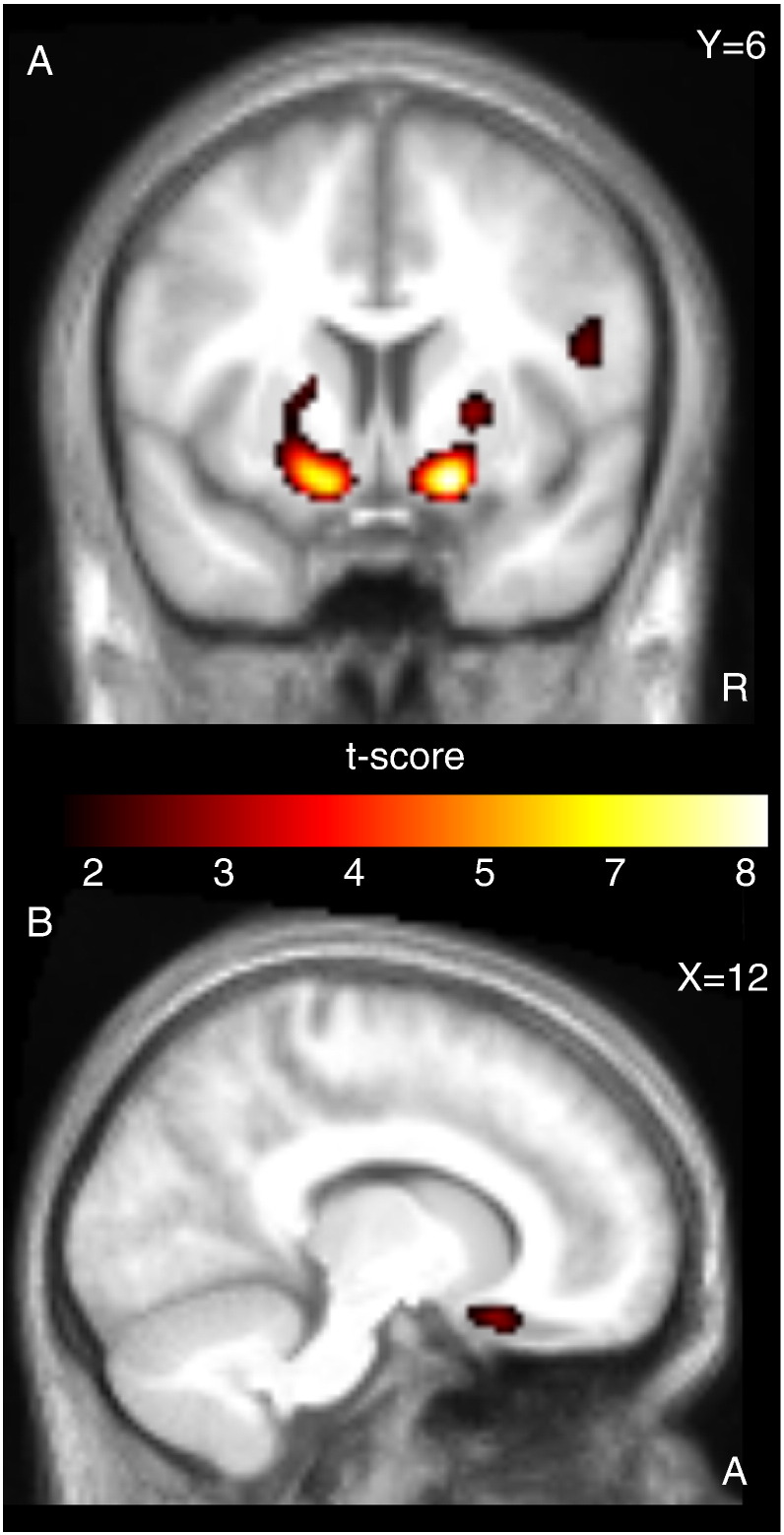
A) Regions where positive outcomes elicited higher BOLD response than negative outcomes (p < 0.05 FWE corrected) across all risk levels. B) Conjunction analyses showing reduced activity in orbitofrontal cortex during both low-risk negative outcomes and high-risk positive outcomes in the ketanserin session relative to the control session (shown at p < 0.01 uncorrected for illustrative purposes).

Positive outcomes (i.e. winning a bet) activated a partially overlapping network of brain regions as for negative outcomes. Yet, when contrasting the BOLD response to positive and negative outcomes, several clusters displayed a significantly stronger response to positive relative to negative outcome activity independently of risk level or drug intervention (Table 1). Notably, the largest clusters showing a stronger activation for positive outcomes were found in VS (Fig. 5A). There were no significant clusters showing higher response to negative outcomes compared to positive outcomes. The group-by-risk ANOVA analysis yielded no significant interaction between the risk level and effect of ketanserin. However, paired tests revealed a cluster in right lateral OFC showing a trend towards an attenuated BOLD response to high positive outcomes caused by high-risk choices when 5-HT_2A_ receptors had been acutely blocked (peak in right OFC at [12 22 − 20], Z = 3.5, p < 0.001 uncorrected). A conjunction analysis revealed that this cluster overlapped with the region showing a reduced BOLD response to low-risk negative outcomes under 5-HT_2A_ receptor blockade (Fig. 5B). Critically, no effects of 5-HT_2A_ receptor blockade could be observed in relation to positive outcomes in the lateral frontopolar region and VS even when applying a liberal statistical threshold (p < 0.01, uncorrected).

### Neural activity during the decision phase

During the decision phase, we found a significant effect of risk magnitude of the choice with a positive linear increase in VS and left inferior parietal lobe response with the risk level (Table 1). There was, however, no significant interaction between the risk level and the ketanserin intervention during the decision phase.

### Mood assessment

The participants completed a Profile of Mood States (POMS) form upon arrival at every investigation day as well as right after the fMRI session. The POMS scores allowed us to identify mood changes caused by the scanning session itself as well as changes related to the administration of ketanserin. An ANOVA analyses including the factors 5-HT challenge (ketanserin vs. no drug condition) and time of measurement (before vs. after the fMRI run), yielded neither a main effect of 5-HT_2A_ receptor blockade nor a challenge × time interaction for any of the reported mood states. We did however find a non-specific time effect with decreased anger (F(19) = 12.2, p = 0.002) and vigor (F(15) = 9.2, p = 0.009) scores and increased fatigue scores (F(15) = 7.6, p = 0.014) at the end of the scanning session compared to upon arrival scores which was unrelated to the pharmacological challenge. Considering the ketanserin session alone, we found an expected decrease in vigor/activity (t(18) = 4.0, p = 0.001), and increase in fatigue/inertia (t(18) = − 3.4; p = 0.004) when contrasting upon *arrival* scores with *end of session* scores. In order to control whether ketanserin's observed effect on risk behavior and BOLD response might have been driven by changes in vigor/activity or fatigue/inertia, we included these POMS values as covariates in the statistical models for behavior and BOLD responses. Critically, the changes in vigor/activity, fatigue/inertia following ketanserin administration did not predict either the increased risk aversion or the altered BOLD response to low-risk negative outcomes reported above.

### Perfusion analysis

We found increased baseline level blood perfusion following ketanserin administration in a widespread prefrontal area including anterior cingulate cortex and inferior frontal regions with right side prevalence (at the significance level accepted for the fMRI analysis). Exclusive masking of our fMRI contrasts with Arterial Spin Labeling (ASL) images revealed that none of the regions showing increased perfusion overlapped with lateral fronto-cortical regions found to be attenuated by ketanserin during negative outcomes or the ventral striatal regions that correlated with the change in risk-taking behavior. We did not find any significant decrease in perfusion levels for the ketanserin session. Since we did not observe any changes in blood perfusion levels in ventrolateral prefrontal cortex and VS we conclude that in these regions the observed attenuation in BOLD response following ketanserin infusion are likely to reflect altered neural activity.

### Comparison between the ketanserin and citalopram sessions

There was no significant difference in risk choice behavior between the citalopram and control sessions. Critically, we were able to replicate the ketanserin induced risk aversion when comparing the ketanserin with citalopram sessions (F1,19 = 10.0, p = 0.005). Substituting the functional images from the control session with the images from the citalopram session revealed similar results, with ketanserin decreasing BOLD response bilaterally in lFPC for low-risk negative outcomes and inverting the linear relationship between negative outcome related striatal activity and risk preference (see Table 2).

## Discussion

Our present data provide direct evidence for a causal link between risk avoidance and 5-HT_2A_ receptor related serotonergic neurotransmission. The increased tendency to “play-it-safe” after 5-HT_2A_ receptor blockade indicates that normal 5-HT_2A_ receptor function tunes behavior towards more risk-taking behavior. Concurrent fMRI measurements revealed that 5-HT_2A_ receptors contribute to the processing of negative outcomes in lFPC caused by low-risk decisions. Further, normal 5-HT_2A_ receptor function is associated with a stronger responsiveness of the VS to low-risk negative outcomes the more risk-seeking the individual behavior is. This relationship was inverted by 5-HT_2A_ receptor blockade. The observed effects were specific to 5-HT_2A_ receptor blockade because they are not observed in general pharmacological manipulations of 5-HT levels (Macoveanu et al., in press).

### Increased risk aversion after 5-HT_2A_ receptor blockade

The increased propensity for low-risk choices under ketanserin suggests that 5-HT_2A_ receptor-related neurotransmission is involved in regulating risk aversion, favoring risky choices, possibly via its facilitatory effects on dopaminergic mesolimbic and nigrostriatal projections (Alex and Pehek, 2007, Boureau and Dayan, 2011). While the propensity to choose the less risky options was influenced by the outcome of the preceding trial in relation to the risk level (either losing a low-risk bet or winning a high-risk bet prompted more risk averse behavior in the following trial), this recency effect was not modulated by ketanserin. Our results suggest that ketanserin exerted a more sustained effect on risk avoidance rather than altering risk choice behavior according to the recent outcome history.

Within the framework of the prospect theory (Kahneman and Tversky, 1979), the 5-HT_2A_ receptor blockade weakened the relative decision weight of low probabilities with high potential reward or strengthened the decision weights of high probabilities with low potential reward, or both. Noteworthy, the potential losses were relatively small as opposed to wins and kept constant across trials. This introduced a “gain frame” that tuned decision making towards “risk aversion for gains” rather than “risk seeking for losses” (Ludvig and Spetch, 2011).

The increased risk aversion after acute 5-HT_2A_ receptor blockade contrasts with the observed effects of global serotonergic challenges. In the same group of subjects, neither dietary ATD nor SSRI intervention affected risk-taking behavior (Macoveanu et al., in press). Interestingly, in that study, the two serotonergic challenges affected different parts of the reward system, namely amygdala and dorsomedial PFC. Global manipulation of 5-HT availability might have a variable impact on a broad range of 5-HT receptors that might have opposing effects on risk-taking and risk-aversion. This could explain the dissimilar effects observed for ketanserin and ATD. Other studies on healthy volunteers have reported conflicting results. ATD was found to either stimulate (Long et al., 2009, Rogers et al., 1999a, Rogers et al., 1999b) or not impact risk-taking behavior (Cools et al., 2008, Rogers et al., 2003).

### Reduced response of lateral frontopolar and orbitofrontal cortex after 5-HT_2A_ receptor blockade

At baseline, a region in left and right lFPC responded to negative outcomes caused by low-risk choices (Fig. 3, right panels). In these instances, a play-it-safe strategy did not pay off as participants missed out on a large reward. These regions did not process negative outcomes following high-risk choices when the missed reward was minimal. Because the size of losses was kept constant across risk choices, the response of the lFPC did not reflect loss magnitude. Although our design did not allow to directly differentiate whether the lFPC reacts preferentially to the magnitude of missed reward or to low-risk negative outcomes, previous neuroimaging work might provide possible clues: while the lFPC has not been found to scale with losses, it has been shown that this region tracks the missed rewards of the best unselected choice and maintains this information across time in favor of their future employment (Boorman et al., 2009, Boorman et al., 2011). Interestingly, patients with orbitofrontal lesions are no longer able to account for the reward of an alternative choice (Camille et al., 2004). We therefore argue that the lFPC processed the magnitude of the missed reward of the alternative unselected choice, when subjects “played-it-safe” but still lost. The magnitude of missed rewards for low-risk options is much higher than the magnitude of missed rewards following high-risk options. This difference in the missed reward may account for the selective behavioral effect of ketanserin on low-risk choices. We suggest that the lFPC generates a 5-HT_2A_ receptor-dependent outcome signal leading to an overall reinforcement of risk-taking behavior. When 5-HT_2A_ receptor signaling is blocked by ketanserin, this reinforcement signal is destabilized, rendering subjects less responsive to high missed rewards, thus tuning the “decision weights” of win probabilities.

OFC is a critical component of the reward network being involved in the evaluation of both rewarding and aversive stimuli (Liu et al., 2007, O'Doherty, 2004). Recent studies have demonstrated that OFC activity is modulated by 5-HT. For instance, increasing 5-HT availability by SSRI treatment of healthy volunteers found decreased neural response to aversive gustatory stimuli in OFC (McCabe et al., 2010). We show that blocking the 5-HT_2A_ receptors leads to an attenuation of the OFC response, but compared to the frontopolar region, the region in OFC responded less to both low-risk negative outcomes and high-risk positive outcomes. We therefore suggest that 5-HT_2A_ related signaling enhances the responsiveness of OFC to “surprising” events, including unlikely positive outcomes with high rewards as well as unlikely negative outcomes with high missed rewards (Schoenbaum et al., 2009).

### Altered relationship between choice behavior and negative outcome processing in ventral striatum

In an fMRI study on the neural correlates of loss aversion, Tom et al. (2007) found that inter-individual differences in behavioral loss aversion predicted inter-individual differences in VS activity coding “decision utility”. The group further showed a negative correlation between the magnitude of potential losses and VS response. Since people prefer avoiding losses to making gains, we suggest that while evaluating possible gains in our task, behavioral loss aversion is linked to behavioral risk aversion. The present results therefore provide new evidence that the evaluation of missed reward caused by low-risk choices in VS also correlates (negatively) with individual risk-averse behavior: in the context of normal 5-HT_2A_ receptor function, large missed rewards caused by low-risk decisions induced weaker VS response in subjects that were relatively more risk averse. Noteworthy, this pattern was not observed in frontopolar areas. We suggest that in normal condition, VS evaluates salient negative outcomes (low-risk) assigning higher value to these events the less frequently they occur (Zink et al., 2004). Because risk-seeking subjects choose low-risk options less frequently, they would show a higher VS response to negative outcomes following low-risk choices compared to risk avoiding individuals. The VS response to these salient events may therefore reinforce risk-seeking behavior. Blockade of the 5-HT_2A_ receptors may reduce the saliency of these events either by means of neuromodulatory action or indirectly, as consequence of the overall increased risk aversiveness.

Previous studies have provided evidence that 5-HT modulates the VS response to primary reward stimuli. For instance, following a seven-day SSRI intervention, Abler et al. (2012) showed reduced VS response to erotic visual stimuli and McCabe et al. (2010) show reduced VS response to pleasant taste. Compared to primary rewarding stimuli like sexual and gustatory stimuli, monetary gains are secondary reinforcers. This might explain why ketanserin did not have a consistent direct effect on VS response to monetary outcomes in the present study. Even though limbic reward areas including VS and OFC have been found to process both primary and secondary reinforcers, there is also evidence that reward-related regions might be stimulus specific (Lamy, 2007). Our data support the view that 5-HT modulation of VS might be limited to primary reward stimuli.

### Methodological considerations

While ketanserin has the highest affinity for the 5-HT_2A_ receptors, it also binds to histamine H1 receptors, and has low affinity binding for 5-HT_2C_ and alpha-1 adrenergic receptors (Glennon et al., 2002, Korstanje et al., 1986). These low affinity interactions with other neurotransmitter systems might have contributed to the observed effects, in addition to an indirect influence of 5-HT_2A_ receptors on dopaminergic neurons (Pehek et al., 2001).

We checked for possible non-specific effects of intravenous ketanserin administration by rerunning the analyses comparing the ketanserin with citalopram session in the same subjects. The behavior and neuroimaging effects of ketanserin that were found in comparison with the control session were fully replicated in a direct comparison against the citalopram challenge. Additional post-hoc analyses verified that ketanserin's effect on mood states and cerebral blood perfusion did not account for the ketanserin-induced task specific changes in BOLD response.

### Conclusion

In conclusion, we suggest that 5-HT_2A_ receptors are involved in the generation of negative outcome signals caused by risk-averse choices, which led to high missed rewards, thereby reinforcing risk-taking behavior. Our findings are of relevance for understanding the role of 5-HT_2A_ receptor signaling in psychiatric disorders. For instance, the 5-HT_2A_ receptors have been linked to personality traits like neuroticism (Frokjaer et al., 2008) and impulsivity (Nomura et al., 2006) and excessive concentration of 5-HT_2A_ receptors in prefrontal cortex has been reported in individuals with major depression (Shelton et al., 2009). Drugs designed to selectively target the 5-HT_2A_ system may therefore benefit patients with specific types of neuropsychiatric disorders. The present results underscore the potential of receptor specific pharmacological manipulations, being able to tap into the distinct function of the different 5-HT receptors. As such, receptor-specific pharmacological challenges complement more general pharmacological manipulations of 5-HT neurotransmission in humans such as dietary tryptophan depletion. Pharmacological fMRI can be used to map the impact of these specific challenges on specific neural networks and hereby, can provide valuable insights into the role of the serotonergic system in reward processing and decision-making.
